# Integrated treatment for autonomic paraneoplastic syndrome improves performance status in a patient with small lung cell carcinoma: a case report

**DOI:** 10.1186/s12883-018-1192-3

**Published:** 2018-11-10

**Authors:** Tatsuya Ueno, Yukihiro Hasegawa, Rie Hagiwara, Tomoya Kon, Jin-ichi Nunomura, Masahiko Tomiyama

**Affiliations:** 1 Department of Neurology, Aomori Prefectural Central Hospital, 2-1-1 Higashi-Tsukurimichi, Aomori, 030-8551 Japan; 2 Department of Respiratory Medicine, Aomori Prefectural Central Hospital, 2-1-1 Higashi-Tsukurimichi, Aomori, 030-8551 Japan

**Keywords:** Autonomic dysfunction, Paraneoplastic neurological syndrome, Small cell lung carcinoma, Anti-ganglionic acetylcholine receptor autoantibodies, Autonomic nervous system diseases

## Abstract

**Background:**

Paraneoplastic neurological syndromes (PNS) are rare disorders associated with cancer and are believed to be immune mediated. Patients with autonomic PNS suffer from variable combinations of parasympathetic and sympathetic failure. Autonomic PNS are usually associated with other PNS, such as encephalomyelitis and sensory neuropathy; however, autonomic symptoms may rarely manifest as PNS symptoms. Autonomic symptoms, therefore, may be overlooked in patients with cancer.

**Case presentation:**

We described a 65-year-old Japanese man who was diagnosed with autonomic PNS due to small-cell lung carcinoma (SCLC) with Eastern Cooperative Oncology Group (ECOG) performance status 3, who suffered from orthostatic hypotension, and urinary retention needing a urethral balloon. Laboratory studies showed decreased levels of noradrenaline, and were positive for anti-ganglionic acetylcholine receptor antibody, type 1 antineuronal nuclear antibody, and sry-like high mobility group box 1 antibody. Nerve conduction evaluations and ^123^I-metaiodobenzylguanidine myocardial scintigraphy showed no abnormalities. Abdominal contrast-enhanced computed tomography revealed marked colonic distention. The patient’s autonomic symptoms resolved following integrated treatment (symptomatic treatment, immunotherapy, and additional chemotherapy) enabling the patient to walk, remove the urethral balloon, and endure further chemotherapy. ECOG performance status remained at 1, 10 months after admission.

**Conclusions:**

Integrated treatment for autonomic PNS may improve autonomic symptoms and ECOG performance status of patients with cancer.

## Background

Paraneoplastic neurological syndromes (PNS) are rare disorders associated with cancer, but are not caused directly by tumor invasion, metastasis or as a consequence of treatment. Their pathogenesis is incompletely understood, but immunological factors are believed to be important. About 3–5% of patients with small-cell lung cancer, 15–20% with thymomas, and 3–10% with B-cell or plasma-cell neoplasms develop PNS [1]. PNS are characterized by classical or non-classical neurological syndromes, and the presence of cancer and onconeural antibodies [1, 2]. Autonomic neuropathies classified as non-classical neurological syndromes of the peripheral nervous system often complicate small-cell lung carcinoma (SCLC) [1, 2], and are associated with anti-neuronal nuclear antibody type 1 (Hu) and anti-ganglionic nicotinic acetylcholine receptor (gAchR) antibody [1–3]. Autonomic failure related to anti-gAchR antibody is also known as autoimmune autonomic ganglionopathy [4]. With regard to onconeural antibodies, two main types of antigenic targets have been described depending on their cellular location; intracellular antigens (i.e. anti-Hu antibody) and cell surface antigens (i.e. anti-gAchR antibody) [1, 5]. Patients with autonomic PNS suffer from variable combinations of parasympathetic and sympathetic failure, such as bladder and rectal disturbance, and orthostatic hypotension (OH) [6]. These autonomic symptoms result in decreased Eastern Cooperative Oncology Group (ECOG) performance status (PS) and lead to out of indication for cancer therapy. Moreover, it may be overlooked in patients with lung cancer because autonomic PNS due to lung cancer is very rare [7]. Therefore, early diagnosis of PNS, immunotherapy and appropriate treatments for PNS are essential for improving the patient’s quality of life.

We herein report a case of autonomic PNS due to SCLC with anti-α3-gAchR, Hu, and Sry-like high mobility group box 1 (SOX1) antibodies, which was improved by integrated treatment (symptomatic treatment, immunotherapy and additional chemotherapy). The patient provided written informed consent for publication of this report.

## Case presentation

A 65-year-old Japanese man was admitted to our department because of OH. He experienced a dry mouth 6 months before consultation. He undertook urinary catheter indwelling owing to urinary retention and noticed constipation 5 months prior to consultation. Four months previously, his primary care physician performed a screening test because he complained of appetite loss and body weight loss of 5 kg. Chest radiographs showed a tumor-like lesion. He was admitted to the Department of Respiratory Medicine in our hospital to evaluate the tumor-like lesion and was diagnosed with extensive disease-small cell lung carcinoma (ED-SCLC) 1 month before consultation. The tumor stage was stage IVA (T1cN2M1b). Following this, he noticed decreased diaphoresis, and suffered from OH. He undertook chemo-radiation therapy (carboplatin, etoposide and thoracic radiotherapy 50 Gy) for ED-SCLC 2 weeks before consultation. However, his daily living activities were restricted due to sustained OH after admission. ECOG PS decreased to 3 points. His medical history included hypertension at 40 years old, diabetes mellitus at 56 years old, and lumbar spinal stenosis at 59 years old. His family history was unremarkable. His medication included magnesium oxide, mosapride, lubiprostone, sennoside, pregabalin, voglibose and mitiglinide.

On consultation, his blood pressure and heart rate in supine position was 124/67 mmHg and 65/min. On standing, his blood pressure was decreased to 69/44 mmHg, and his heart rate was increased to 88/min. Physical examinations were normal. Neurological examination revealed no limb weakness, ataxia, and sensory disturbance. Pupil size and light reflex were normal, and the other cranial nerve examination was also normal. Deep tendon reflexes were in the normal range and plantar responses were flexor. However, he complained of autonomic nervous system impairment; dry mouth, urinary retention, constipation, decreased diaphoresis, and OH.

**Fig. 1:**
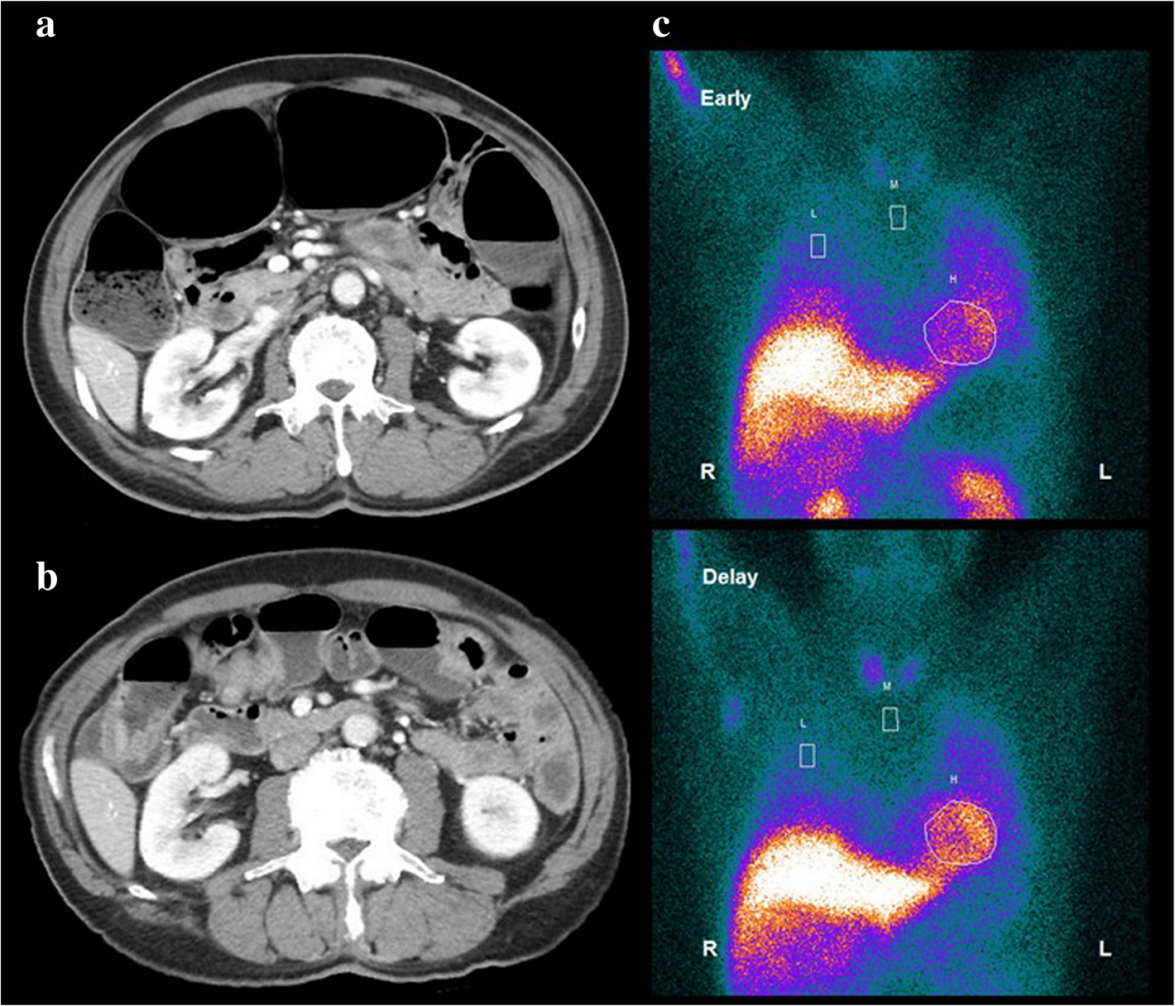
Abdominal contrast-enhanced computed tomography 13 days (**a**) and 83 days (**b**) after admission and ^123^I-metaiodobenzylguanidine (MIBG) myocardial scintigraphy 43 days after admission (**c**). Abdominal contrast-enhanced computed tomography showed marked colonic distention (**a**), and abdominal distention disappeared (**b**). The early and delayed heart/mediastinum (H/M) ratio of MIBG was 2.9 and 4.0, respectively (**c**). The washout ratio was 20.1%

Laboratory evaluations showed elevated levels of fasting blood glucose (147 mg/dl) and hemoglobin A1c (7.2%). Anti-nuclear antibody, rheumatoid factor, anti-Ds-DNA antibody, anti-Sm antibody Anti-SS-A/Ro and anti-SS-B/La antibody tests were all negative. The coefficient of variation of RR interval was decreased (0.72%). 24-h urine catecholamine excretion showed normal level of adrenaline (4.5 μg/day; reference range 3.4–26.9) and dopamine (388.9 μg/day; reference range 365.0–961.5), and decreased level of noradrenaline (11.3 μg/day; reference range 48.6–168.4). Serum catecholamine levels also revealed normal levels of adrenaline and dopamine, but decreased levels of noradrenaline (30 pg/ml; reference range 100–450). Cerebrospinal fluid (CSF) analysis revealed normal cell counts (1 cells/μl), a total protein level of 34 mg/dl, and a glucose level of 94 mg/dl, with a concomitant blood glucose level of 147 mg/dl and a normal IgG index (0.65). Oligoclonal bands were negative. Motor and sensory nerve conduction evaluations showed no abnormalities and both waning and waxing were not shown by the repetitive nerve stimulation test. Abdominal contrast-enhanced computed tomography (CT) revealed marked colonic distention (Fig. 1a). Brain magnetic resonance imaging (MRI) and spinal MRI findings were unremarkable. ^123^I-metaiodobenzylguanidine (MIBG) myocardial scintigraphy was normal (Fig. 1c). Paraneoplastic autoantibodies for Hu and SOX1 were positive. Furthermore, an anti-gAchR antibody test (anti-α3 AchR) was positive. Other paraneoplastic autoantibodies including the anti-neuronal nuclear autoantibodies type 2 (Ri), delta/notch-like epidermal growth factor-related receptor (Tr), glutamic acid decarboxylase 65 (GAD65), zinc finger protein 4 (ZIC4), titin, recoverin, paraneoplastic antigen Ma2 (PNMA2), collapsin-response mediator protein 5 (CRMP or CV2), Purkinje cell antibody type 1 (Yo) were all negative. Detection of anti-α3-gAchR and the other PNS autoantibodies was performed by radioimmunoassay methodology and immunoblot analysis, respectively. Based on these results, we diagnosed the patient with autonomic PNS due to SCLC.

**Fig. 2:**
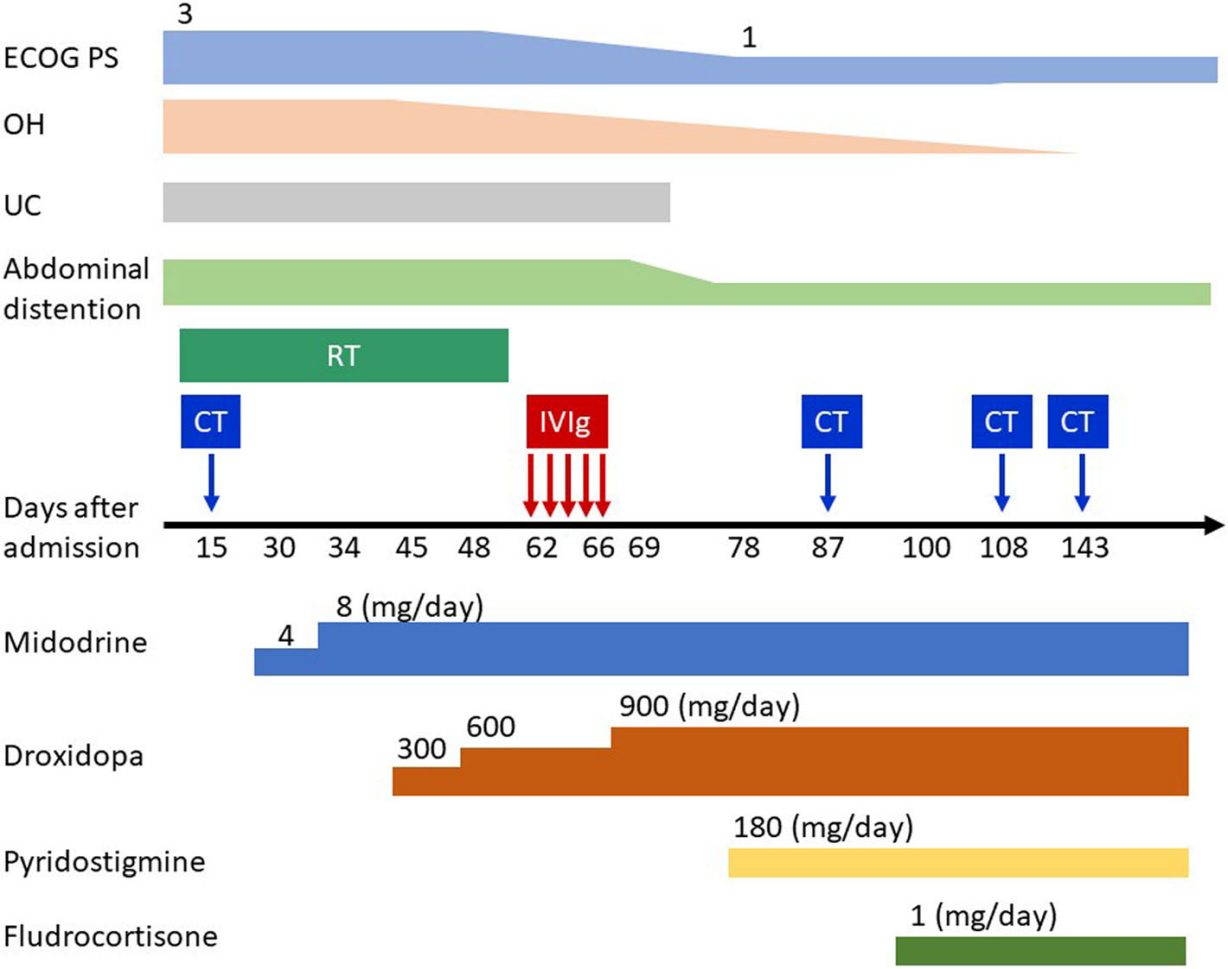
The clinical course of the patient. The dosage of IVIg was 400 mg/kg body weight daily for 5 days. Chemotherapy included carboplatin (5AUC) and etoposide (80 mg/m^2^, 3 days), and radiation therapy was performed by thoracic radiotherapy 50 Gy. AUC: area under the blood concentration time; CT: chemotherapy; ECOG PS: Eastern Cooperative Oncology Group performance status; IVIg: intravenous immunoglobulin; OH: orthostatic hypotension; RT: radiation therapy; UC: urethral catheter

We used midodrine (8 mg/day), droxidopa (900 mg/day), pyridostigmine (180 mg/day), and fludrocortisone (0.1 mg/day) to treat the OH, and intravenous immunoglobulin (IVIg) 400 mg/kg body weight daily for 5 days because of diabetes mellitus and a catheter-associated urinary tract infection during admission (Fig. 2). These treatments improved his autonomic symptoms due to PNS. He was able to walk again without the symptoms of OH, and ECOG PS improved to 1 point. Amelioration of urinary retention meant that the urethral balloon could be removed. Abdominal distention disappeared, confirmed by an abdominal CT after 83 days (Fig. 1b). The patient was then able to receive the 3 remaining courses of chemotherapy as his ECOG PS improved, and he was discharged 101 days after admission. On day 43 after discharge, his blood pressure in supine and standing positions were 124/83 mmHg and 117/69 mmHg, respectively. Four-course chemotherapy for SCLC achieved a partial response. The autonomic symptoms had not recurred and his ECOG PS remained at 1 point, 10 months after admission.

## Discussion

We have herein presented the autonomic PNS associated with anti-α3-gAchR, Hu and SOX1 antibodies in a patient with SCLC. This case suggests that symptomatic treatment, immunotherapy, and additional chemotherapy for autonomic PNS may improve the autonomic symptoms and performance status of patients. If patients with cancer suffer from autonomic symptoms, oncologists and neurologists should consider the possibility of autonomic PNS even in advanced cases.

Autoantibodies involved in autonomic PNS include Hu, Yo, CV2, PCA-2, GAD-65, voltage-gated calcium channel (VGCC) and gAchR antibodies [6]. Autonomic PNS are usually associated with other PNS, such as encephalomyelitis and sensory neuropathy [6]. Patients with SCLC rarely present with chronic gastrointestinal pseudo-obstruction and OH without other neurological disturbances caused by anti-Hu antibodies [8, 9]. Autonomic symptoms, therefore, may only manifest as PNS symptoms [8, 9]. In this case, the patient had three autoantibodies for Hu, gAchR, and SOX-1. Autonomic symptoms were only detected after neurological examination and laboratory tests, and the patient had no other neurological symptoms, including sensory symptoms. With regard to autonomic symptoms, anti-Hu antibodies usually cause chronic gastrointestinal pseudo-obstruction or acute pandysautonomia as part of encephalomyelitis or subacute sensory neuropathy [6, 7, 10, 11], whereas anti-gAchR antibodies are mainly associated with subacute pandysautonomia [1, 4, 6]. Anti-SOX-1 antibodies are also detected in Lambert-Eaton syndrome with SCLC [12, 13]. SOX1 reactivity is predominantly associated with anti-Hu antibodies and SCLC [14]. Anti-Hu and SOX-1 antibodies target intracellular antigens [1, 5, 12], while anti-gAchR antibodies target cell surface antigens [1]. PNS related to antibodies for intracellular antigens respond poorly to immunotherapy; however, PNS related to antibodies for neuronal cell surface antigens usually respond well to immunotherapy [5]. The patient described herein presented with only autonomic symptoms without other PNS symptoms, along with decreased levels of 24-h urine and serum catecholamine excretion and normal MIBG scintigraphy assessing the sympathetic cardiac nerve terminals. This suggested that autonomic ganglia of the sympathetic nerve were involved. Although symptomatic treatment and chemotherapy for SCLC had an effect on the recovery of autonomic symptoms, IVIg markedly ameliorated the OH, abdominal symptoms, and urinary retention. These results demonstrate that the autonomic symptoms of this patient were caused by antibodies to cell surface antigens, especially anti-gAchR antibodies. About 15% of patients have paraneoplastic autoimmune autonomic ganglionopathy usually associated with SCLC or thymoma [15]. However, in Japanese patients with autoimmune autonomic ganglionopathy, 10% patients had ovarian tumors, pancreas cancer, mediastinal tumors, and paranasal cancer, but not SCLC [4]. Paraneoplastic autoimmune autonomic ganglionopathy due to SCLC associated with anti-gAchR antibodies are likely overlooked in Japan.

The management of PNS requires not only immunotherapy but also oncological treatment [5]. Our patient suffered from autonomic symptoms and his ECOG PS declined. If the cause of the autonomic symptoms were not identified, the patient would have lost the opportunity to receive the additional chemotherapy. Survival from time of diagnosis is 7 month (median) in patients with anti-Hu antibodies [16]. Anti-gAchR antibodies and anti-Hu antibodies often coexist in patients with paraneoplastic autonomic neuropathy due to SCLC [17]. Our patient received the symptomatic treatment, immunotherapy, and additional chemotherapy. As a result, our patient was still alive 10 months after admission, and his ECOG PS remains 1 point. Compared with PNS due to anti-Hu antibodies, this case report highlights the improvement in ECOG PS and that ECOG PS could be maintained for 10 months because of the integrated treatment regime [16]. Furthermore, this case is unique in that the autonomic symptoms responded well to the integrated treatment, despite antibodies for intracellular antigens and neuronal cell surface antigens being simultaneously detected. Even in cases positive for anti-Hu antibodies, if neurological symptoms are only autonomic symptoms, the effects of other PNS related antibodies (i.e. anti-gAchR antibodies) should be considered, and immunotherapy ought to be performed.

## Conclusion

We present a patient with autonomic PNS due to SCLC whose symptoms were improved by integrated treatment; symptomatic treatment, immunotherapy, and additional chemotherapy. Integrated treatment for autonomic PNS may improve the autonomic symptoms and ECOG PS of patients. If patients with cancer suffer from autonomic symptoms, physicians should differentiate autonomic PNS even in cases of advanced tumor stage, and integrated treatment may improve patients’ survival outcome.

## Abbreviations

CRMP or CV2: Collapsin-response mediator protein 5
CSF: Cerebrospinal fluid
CT: Computed tomography
ECOG: Eastern Cooperative Oncology Group
ED-SCLC: Extensive disease-small cell lung carcinoma
gAchR: ganglionic nicotinic acetylcholine receptor
GAD65: Glutamic acid decarboxylase 65
Hu: Neuronal nuclear antibody type 1
IVIg: Intravenous immunoglobulin
MIBG: 123I-metaiodobenzylguanidine
MRI: Magnetic resonance imaging
OH: Orthostatic hypotension
PNMA2: Paraneoplastic antigen Ma2
PNS: Paraneoplastic neurological syndromes
PS: Performance status
Ri: Neuronal nuclear autoantibodies type 2
SCLC: Small cell lung carcinoma
SOX-1: Sry-like high mobility group box 1
Tr: delta/notch-like epidermal growth factor-related receptor
VGCC: Voltage-gated calcium channel
Yo: Purkinje cell antibody type 1
ZIC4: Zinc finger protein 4
